# Use of Local Intelligence to Reduce Energy Consumption of Wireless Sensor Nodes in Elderly Health Monitoring Systems

**DOI:** 10.3390/s140304932

**Published:** 2014-03-11

**Authors:** Thomas J. Lampoltshammer, Edison Pignaton de Freitas, Thomas Nowotny, Stefan Plank, João Paulo Carvalho Lustosa da Costa, Tony Larsson, Thomas Heistracher

**Affiliations:** 1 School of Information Technology and Systems Management, Salzburg University of Applied Sciences, Urstein Süd 1, Puch/Salzburg 5412, Austria; E-Mails: tnowotny.lba@fh-salzburg.ac.at (T.N.); stefan.plank@sbg.at (S.P.); Thomas.Heistracher@fh-salzburg.ac.at (T.H.); 2 Department of Applied Computing, Federal University of Santa Maria, Santa Maria 97105-900, Brazil; E-Mail: edison.p.freitas@ufsm.br; 3 Laboratory of Array Signal Processing, Department of Electrical Engineering, University of Brasilia, Campus Universitário Darcy Ribeiro, S/N, Asa Norte, Brasília 70910-900, DF, Brazil; E-Mails: edisonpignaton@unb.br (E.P.F.); joaopaulo.dacosta@ene.unb.br (J.P.C.L.C.); 4 School of Information Science, Computer and Electrical Engineering, Halmstad Universtity, Kristian IV:s väg 3, Halmstad 301 18, Sweden; E-Mail: tony.larsson@hh.se

**Keywords:** sensors, wireless, energy, AAL, health care

## Abstract

The percentage of elderly people in European countries is increasing. Such conjuncture affects socio-economic structures and creates demands for resourceful solutions, such as Ambient Assisted Living (AAL), which is a possible methodology to foster health care for elderly people. In this context, sensor-based devices play a leading role in surveying, e.g., health conditions of elderly people, to alert care personnel in case of an incident. However, the adoption of such devices strongly depends on the comfort of wearing the devices. In most cases, the bottleneck is the battery lifetime, which impacts the effectiveness of the system. In this paper we propose an approach to reduce the energy consumption of sensors' by use of local sensors' intelligence. By increasing the intelligence of the sensor node, a substantial decrease in the necessary communication payload can be achieved. The results show a significant potential to preserve energy and decrease the actual size of the sensor device units.

## 1. Introduction

The elderly population in many European countries is increasing. This is due to decreasing birth rates, increasing living and medical standards, coupled with higher incomes for the group of above 60 years to that they can afford more healthy food and perform stimulating activities from which their health benefits [1,2]. In order to help with the process of all day independence for the elderly people, several home care institutes and supporting companies, as well as governmental programs, provide a suitable environment and treatment for them. According to health care companies and home care institutions, injuries from falls represent the major health issue among elderly people. Falling represents a severe issue for elderly people and has a prominent position among health care issues due to its frequency and the associated health care costs [3]. Besides possible deaths caused by falls, other related concerns are non-fatal injuries [4], fear of falling again [5] and the loss of an independent life [6].

Ambient Assisted Living (AAL) is a concept or framework aimed at supporting elderly people in their desire to retain autonomy and assisting them in their activities of daily living (ADL). An established approach to designing AAL systems is to integrate omnipresent devices into people's environment to increase their safety and their well-being [7]. However, it should not be forgotten that elderly people usually tend to be afraid of new technology and therefore are prone to avoid its uptake. In addition to user-friendly interfaces, the introduced technology should be as non intrusive as possible [8]. One solution to this issue is the miniaturisation of the devices used. For the detection of falls, wireless, wearable body sensors can be employed. In order to function properly and independently over a long period of time, the energy-storing capacity of the batteries of such devices plays a major role. In order to provide a long lifetime, coupled with a small size, the energy consumption of the embedded sensors becomes even more crucial. In this paper, we propose an approach for the use of local intelligence to reduce energy consumption of wireless sensor nodes in elderly health monitoring systems. By increasing the intelligence of the sensor node, a substantial decrease in the necessary communication payload can be achieved. This payload reduction leads to substantial reduction of the energy consumption per sending process. The energy savings from this proposed approach can be used to decrease the actual size of the sensor device units or to allocate the freed capacity to additional components within the sensor device.

The remainder of the paper is organised as follows: after the related work towards energy consumption in wireless sensors and AAL is presented in Section 2, Section 3 discusses the AAL scenario for this paper, together with in-depth details about the method employed in fall detection, the sensor intelligence approach, as well as the sensor threshold chosen to describe the rapid body movement during falls. Subsequently, the test-bed results are presented and discussed in Section 4. Section 5 closes the paper with some conclusions and an outlook.

## 2. Related Work

Sensor platforms play an import role in the application area of AAL. For instance, Wille *et al.* [9] introduced a concept for a generic, reusable sensor platform called *TinySEP* to provide an open platform for AAL developers. Amoretti *et al.* [10] proposed a concept for data fusion for sensors in AAL to provide context awareness to react on user activity. Especially in applications of monitoring in the health sector, AAL has been the focus of various research endeavours. To monitor activities in an elderly person's living environment, sensor networks in the living space paired with visual-based permanent surveillance, e.g., cameras, are one possible solution. For example, Aghajan *et al.* [11] employ a multi-visual device setup to analyse a person's body posture before, during or after an alert occurred in the system to assess the overall situation. However, such approaches confront all involved persons with severe privacy issues [12].

The use of wireless sensors is providing a less intrusive way to monitor peoples' ADLs and helping medical personal to recognise patterns of emerging diseases and unhealthy habits. Therefore, the main focus of interest in the presented study rests on the body-carried sensor alternative. In the work of Han *et al.* [13], a framework for monitoring lifestyle diseases using a wireless sensor is presented. The goal of their work is to identify early symptoms of diseases such as depression or Alzheimer's based on long-term activity monitoring. This task is performed by providing the monitored persons with wireless sensors that are set to acquire specific data indicating patterns that can be recognised by a lifestyle disease predictor as possible symptoms of a given disease. Shin *et al.* [14] proposed to monitor the elderly living alone aiming to detect abnormal living patterns. In their work, the data acquired by sensors are handled as vector data descriptions to provide inferences that make possible the detection of patterns that diverge from the normal behaviour. These patterns indicate a early stage of diseases, similarly to what is proposed in [13]. The work presented in [13,14] concentrates on the framework itself, but without special concern on the technical details involved in the sensors themselves and in the network formed by them. Instead, Custodio *et al.* [15] focussed exactly on the technological advances in wireless sensor devices usable in health care monitoring systems. They discuss the main aspects involved in the usage of wireless sensors in u-health (ubiquitous health) applications, *i.e.*, communication technologies, sensors' dimensions, energy resource usage, among others. For a comprehensive review of wireless body sensor networks for health-monitoring applications, please refer to [16].

Focusing specifically on the energy concern, various research works concentrate on the issue of energy consumption of wireless sensor devices. Györoe and Pataki [17] present a method for measuring and optimising the process of scheduling within wireless sensor networks based on hidden Markov models. Another example is given by the paper of Zhang *et al.* [18]. It discusses the concept of using two transmission channels instead of one - this is based on the hypothesis that it is often not necessary to keep a transmission channel continuously alive and that it is sufficient to trigger data transmissions from the sensor node on-demand only. The introduced second channel is dedicated to surveillance purposes. Thus it enables the sensor node to provide transmission on-demand capabilities combined with zero energy consumption during idle time. With respect to the second channel, only a receiving unit is required. Such a receiving unit can be realised by installing a radio-frequency identification (RFID) module on the sensor node.

In return, the RFID offers benefits in terms of instant response as well as energy scavenging and no idle energy consumption while listening. On activation, the RFID module initialises the regular transmission module and the gathered data of the sensor can be transmitted via the first channel. Mondal *et al.* [19] proposed a reduction of the payload of the transmissions via the application of compression techniques like linear predictive coding (LPC). In order to prove their point, the authors conducted a comparison between this method and others as well as plain transmissions.

The proposition of this paper also aims at the reduction of the number of transmissions. Though not based on compression techniques, the sensor node's intelligence is altered to reduce the communication payload instead.

Reduction of energy consumption is not the only possible way to increase a sensor node's lifetime. As mentioned before, there is great potential in energy harvesting as well. An on-going development shows interest not only in green energy from solar energy collectors, but rather in body related energy sources as well. Examples for this is the work of Renaud *et al.* [20] or Lauterbach *et al.* [21] and Lenonov *et al.* [22] which is focussing on gaining energy by exploiting the motion of the human body or its thermal energy. Photovoltaic energy harvesting alternatives for indoor applications are also considered, as explored in [23], which describes a system using indoor dispersed photovoltaic sources for energy harvesting to charge batteries of cell phones.

## 3. Experimental Setup

This section describes the overall setup of the experiment. In more detail, we introduce in Section 3.1 the setup for the AAL environment. Section 3.2 focuses on the fall detection, while Section 3.3 treats the three levels of sensor intelligence applied in our approach. Subsequently, Section 3.4 discusses the threshold setup for sensor logic *L2*, followed by Section 3.5, presenting the machine learning approach for sensor logic *L3*.

### 3.1. Setting up the AAL Environment

**Figure 1.**
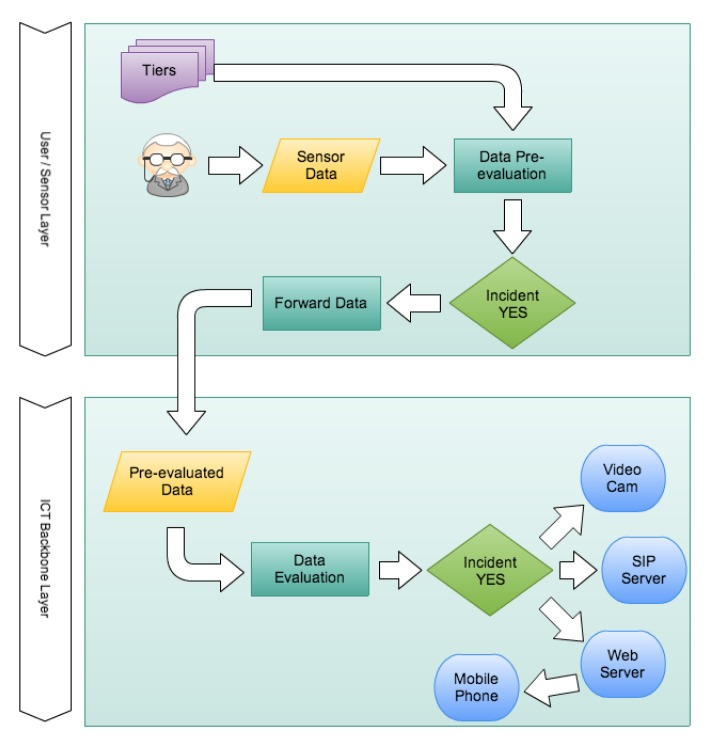
Proposed approach workflow.

**Figure 2.**
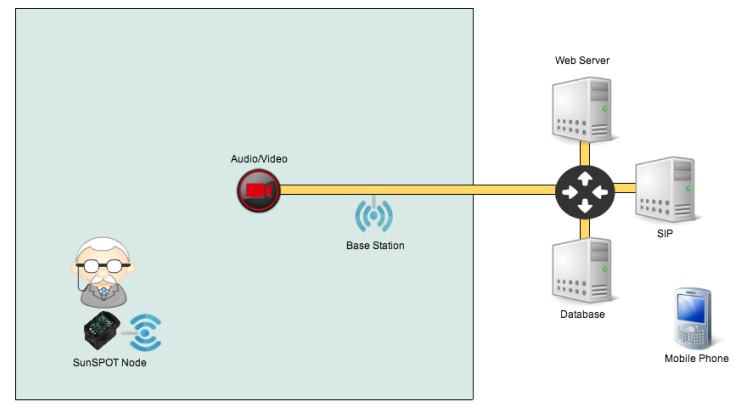
Schematic of test-bed environment.

The AAL environment aims at the simulation of an ambulant care system, designed to support elderly people in their ADLs in their homes or at residential flats in retirement homes. In the following part, the process flow (Figure 1) is described, while the specific components mentioned in the description can be seen in Figure 2. The user moves within an adapted environment. As a wearable body sensor, the user is equipped with a wireless sensor implemented by an Oracle SunSPOT node [24].

These sensor nodes contain, besides other sensors such as light sensors or temperature sensors, a tri-axial accelerometer. This accelerometer is used to detect potential falls. The data gathered from the SunSPOT node are, depending on the intelligence level activated, constantly evaluated. If a possible fall is detected, the SunSPOT node transmits observation data to its base station.

In the base station, the received data can then be processed for an in-depth analysis to prove or disprove the pre-evaluation results from the sensor or, in case of a higher intelligence, just react accordingly. If an actual fall has occurred, the systems can activate bi-directional communication devices (audio and video).

The Session Initiation Protocol (SIP) server acts as gateway to an external service provider in order to establish a communication link an external device such as a mobile phone. Finally, the Web server offers configuration and maintenance capabilities for the care personnel. In the test-bed, a critical situation that could occur during ADLs of an elderly person is simulated. If the person falls, the sensor will detect the fall and inform the backend system about the incident. The system will then establish a connection to an audio/video device, so the care personnel can contact the person to ask what happened. In this particular case, the call is relayed to a mobile phone.

We point out here that the use of audio and video recordings, paired with pattern analysis of movements may be abused to violate the privacy of an individual roaming within the described scenario. This issue is intensively and controversially discussed within literature [25]. Thus, this issue has to be handled carefully and needs to incorporate all affected persons. However, at some point it can be necessary to allow provisional privacy violation in order to save lives and to guarantee full functionality of the installed system [26].

As the described environment serves as test-bed only and the main focus of this paper is on the energy consumption of the utilised sensor nodes, this issue is not pursued any further at this point.

### 3.2. Setting up the Fall Detection

Although the detection of a fall is not the main focus of this paper, it still represents an important factor in the ADL environment. Therefore, the authors adapted the approach presented by Bourke and Lyon [27] to identify and set up a reliable and suitable threshold for triggering the fall detection alarm. The sensor is mounted on the user's waist in front of the body in a position compareable to a belt buckle's one. This specific position has been chosen according to the findings of Maurer *et al.* [28]. They analysed the recognition and monitoring accuracy of wearable sensors in different body positions. Their research indicates that for physical activities such as running or standing, the positioning of a sensor on the waist provides high accuracy results throughout various activity patterns. To decide which type of fall—direction and starting position—should be chosen, we based our assumptions upon the following research: Lord *et al.* [29] state that about 82% of all falls happen from a height associated to a standing position.

In regard to the direction of falls, there exist various opinions and research results: O'Neil *et al.* [30] claim in their study that 60% of the falls that elderly people face are forward-directed falls. Hsiao and Robinovitch [31] state that over all ADLs, the most likely fall pattern would be backward-directed falls. For our purpose, *i.e.*, to study the energy saving aspects, we decided to simulate forward-directed falls while standing.

In order to avoid false alarms based on ADLs, the test subjects were monitored throughout the day for several hours. In comparison to the study of Bourke and Lyon [27], we did not discover an overlap of ADLs and the fall threshold for this specific sensor type. As an alternative approach towards the threshold-based implementation, we propose a machine learning-based approach. For the training of this approach, the same sensor data from the test subjects were used as within the threshold-based setup.

### 3.3. Comparing Three Levels of Sensor Intelligence

According to [32], the communication components of a sensor node are in general the highest energy consumers among all components. The respective energy consumption for processing data was found to be negligible in their setup. This finding is supported by an observation of [33]. They state that the processing of several bits consumes less energy than the transmission of a single bit. The main idea of the local processing approach is to exploit the circumstance that usually about 80% of a sensor's energy consumption is related to transmission operations [34].

**Figure 3.**
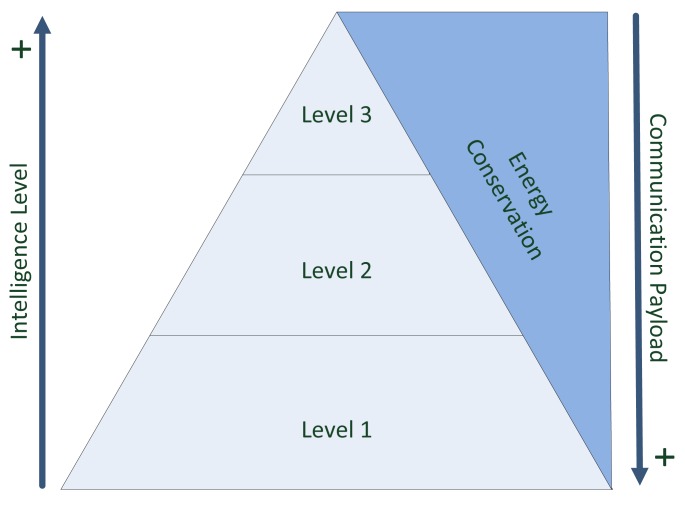
Location of intelligence versus communication payload.

In consequence, a decrease of transmissions by reducing the amount of data to be sent should result in a significantly reduced energy consumption. Hence, to reduce the overall energy consumption, we suggest a local intelligence approach performing significant local data processing (Figure 3).

*L1* can be described as a simple “unintelligent” sensor node. Each measurement in this approach is directly transmitted to the associated base station. The process of identification of a fall is outsourced to the base station and its algorithms implemented there. According to the above-stated hypothesis, this approach should exhibit the highest energy drain in comparison to the other design alternatives.

*L2* represents a “semi-intelligent” sensor node. Sensor nodes on this intelligence level can decide on their own whether data should be transmitted or not. This decision is based on the person's state. Two main states are defined: (i) non-standing; and (ii) standing. While non-standing refers to a person sitting or resting, standing describes a person moving or exercising. During the standing state, the sensor transmits all measurements directly to the base station.

However, during the non-standing state, the sensor stops its transmissions. As all measurements are buffered within the sensor and the actual values are continuously monitored and evaluated in regard to the state detection, no measurements are lost and potential fall indicators (expressed by rapid movement) can still be transferred and detected at the base station. This level of intelligence should show a reduction of the energy consumption as the amount of data was reduced in comparison to *L1*.

Finally, *L3* is the most intelligent variation of the designs compared. It employs a neural network for the detection of incidents. Herewith it becomes possible to use each axis from the accelerometer individually as an input parameter. This setup enables the system to also cover non-linear classifications. As *L3* only sends the classification results of the associated movement pattern, the energy consumption is supposed to be the lowest of all three presented design alternatives.

### 3.4. Defining the Sensor Node Threshold

The decision whether to transmit data or not within the sensor nodes for *L2* relies on a threshold-based approach. Such approaches, incorporating tri-axial accelerometers, are common in the domain of fall detection [35]. Even though the threshold-based approach is a straightforward one, it is still possible to achieve reasonable results with a fall detection accuracy of over 80% [36]. For a comparison study of low-complexity algorithms for fall detection please refer to [37].

The calculation for the actual thresholds for the present publication was performed according to the work of [38]. The Euclidian distance of all vector elements including static and dynamic vector elements was calculated via Equation (1), based on the sensor node's accelerometer measurements. The elements *a*_x_, *a*_y_, and *a*_z_ represent the acceleration in the respective *x*-, *y*-, and *z*-axes.

(1) $$\left|a\right|=\sqrt{{\left(a_{x}\right)}^{2}+{\left(a_{y}\right)}^{2}+{\left(a_{z}\right)}^{2}}$$

**Figure 4.**
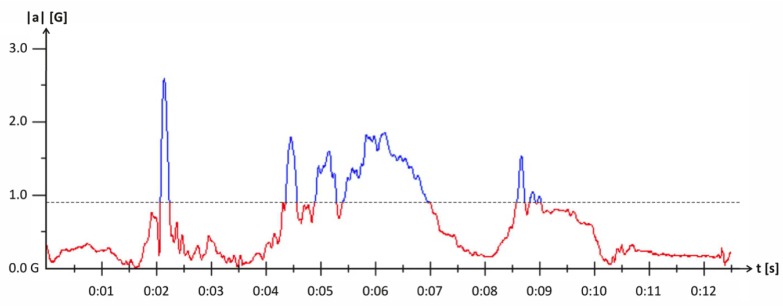
Thresholds for level 2 architecture.

The actual threshold (threshold range) chosen to be employed in *L2* can be seen in Figure 4. The red values (beneath the dashed line) indicate measurements that fall into the category of a non-standing state in *L2*, while the blue values (above dashed line) indicate measurements of a standing state respectively.

### 3.5. Machine Learning-Based Approach

While *L2* is only capable of classifying the incoming values into two classes, *L3* is capable of detecting patterns within the accelerometer values. This is achieved by employing a Multi-layered Perceptron (MLP) [39]. This construct belongs to the family of neural networks and supports the classification of several linear and non-linear input parameters. In particular, the authors utilise a Feed-forward MLP featuring a back-propagation learning algorithm [40]. As underlying patterns, we defined: (i) no movement/walking; (ii) running; (iii) jumping; and (iv) falling.

In contrast to the approach applied within the threshold-based design, every axis of the accelerometer directly influences the neural network. Each axis provides individual input parameters within the input layer. The classification results are presented within the output layer. In order to successfully train the MLP (meaning that the weights before and after the hidden layer are adjusted properly for the specific dataset), all movements that should be detected have to occur sufficiently often during the training phase [41].

**Figure 5.**
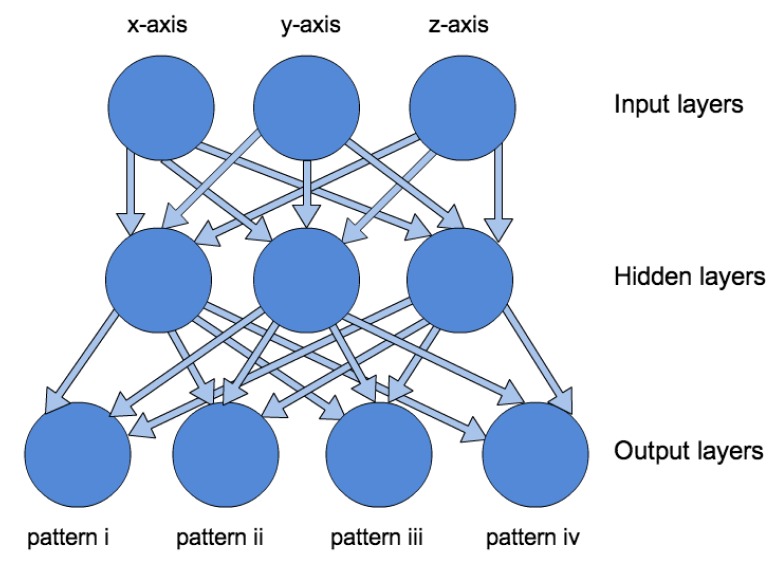
Structure of the employed Multi-layered Perceptron (MLP).

In our case, we used the acceleration in the respective *x*-, *y*-, and *z*-axes for the input layer (see Figure 5). The hidden layer represents the weights which are adjusted during the training phase. For the training, we used data gained from the test subjects. It is worth mentioning that training with data from only one person leads to twofold results. On the one hand, the sensor is specifically trained to the motions of the person wearing the sensor. On the other hand, the same trained experience within the sensor can cause issues if applied to sensors worn by other people, as false alarms or—even worse—non-detected falls could occur.

## 4. Results and Discussion

**Figure 6.**
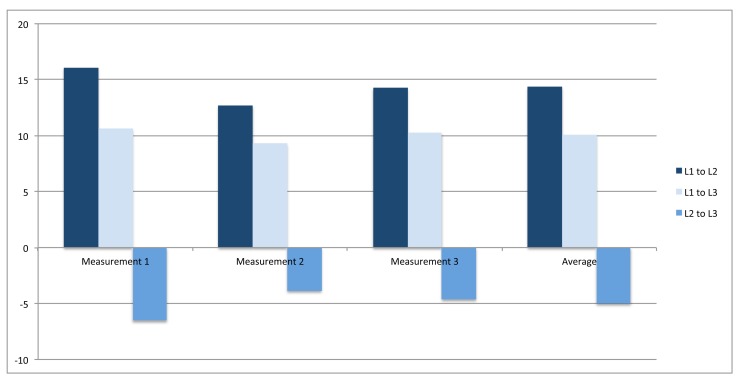
Energy savings of all intelligence levels.

To compare the energy savings of all three local intelligence levels, we continuously measure the electric discharge in μAh for every sending iteration of sensor measurements. The voltage during the simulations for all three local intelligence levels is kept constant at 3 V. Furthermore, no sleep modes are used in the devices so that the energy consumption is proportional to the use of current and time. We conducted three simulation rounds per intelligence level, where each round represents the average sending duration and the average discharge for 1000 sending iterations. When comparing the results of *L1* and *L2* (Tables 1–3), a decline regarding the energy consumption can be seen. Furthermore, if the simulation runs are conducted over the lifetime of the battery, the accompanying reduction of the communication payload becomes obvious (Tables 4–6). *L2* consumed about 14% less energy than *L1* (see Figure 6, right-most average columns). As expected, this can be traced back to the reduction of the transmitted communication payload. This reduction is also reflected within the decrease of the average sending duration.

An interesting fact from the transition from *L2* to *L3* (Tables 2 and 3) is the amount of energy that can be saved. The energy consumption in *L3* is increased in contrast to the transition from *L1* to *L2*. Based on the hypothesis, this behaviour was unexpected. However, as it was demonstrated in previous research [42], a significant energy drain can be observed during idle and overhearing periods. This drain can be as high as transmitting or receiving data [42]. Therefore, it can be stated that it is not possible to reduce the overall energy consumption related to the radio device below a certain value. In addition, for the hypothesis it was assumed that the trade-off in regard to the introduction of the intelligence level *L2* and *L3* would be still in favour of energy savings. However, the negative results (see Figure ) indicate that the calculations performed by *L3* eliminate the energy savings resulting from the reduced communication payload.

When comparing the results of our approach to results from previous research works, although being influenced by the computational load of the intelligent levels, they still present a remarkable amount of energy savings. Cho *et al.* [43] achieved in their work savings up to 30%. Though their results are impressive, they were gained by developing a sensor platform from scratch. Our approach presented in the present work is generic in principle and thus can be deployed on any given platform. Xiao *et al.* [44] were able to obtain savings between 9% and 25%. The arising variations within their approach are based on different restrictions or relaxations in regard to the desired level of detection accuracy. *L2* and *L3* place themselves close to these numbers and achieved competitive results in relation to the conservative settings of Xiao *et al.* In relation to the aggressive setup presented by Xiao *et al.* [44], none of the approaches can compete but this is due to a noticeable reduction in detection accuracy compared to our approach.

Guo *et al.* [45] proposed a Minimum Energy Packet Forwarding Protocol (MEPFP) in combination with a smart Automatic Repeat reQuest (ARQ) procedure. By combining these two components, they achieved a corresponding 12% of saved energy, which is close to the value of energy reductions presented in this paper.

## 5. Conclusions and Outlook

This paper analysed the effects of different levels of local intelligence to reduce energy consumption of wireless sensor nodes used to monitor the health of elderly people. The preferred approach exploits the fact that most energy within wireless sensor nodes is consumed by transmission-related operations.

Therefore, the demonstrated approach aims at reducing the overall amount of data that has to be transmitted. The local processing-based intelligence resulted in about 14% energy savings. This savings level can compete with other research works' results.

It is noteworthy that the proposed local intelligence approach is embedded in a major framework to process the acquired data from the sensor nodes, providing high-level services in an elderly person monitoring system. The presented framework includes the activation of additional devices, e.g., audio and video, and a gateway to provide data to health care personnel upon a detected fall.

Future work is possible mainly in two directions: The first is related to the framework as a whole, which can consider the usage of additional sensors to increase the overall accuracy of the system. These additional sensors could be dedicated to blood pressure or cardio rate, or the adoption of a multi-patient control, which can be used in hospitals or retirement clinics.

Regarding the energy concern, possible further studies can be performed deploying multiple sensor nodes with different offsets regarding the sampling rate. Still related to energy saving, the usage of different frequency bands and communication technologies can also be explored, such as the use of cooperative multiple input multiple output (MIMO) to diminish the energy consumption due to the sensors' communication, as discussed in [46] in cases in which more than one user share the same environment. Furthermore, compression techniques and packet fragmentation could be employed to optimise the payload distribution within the packets for low-energy radio devices.

## Figures and Tables

**Table 1.** Energy statistics *L1*–*L3* for measurement 1.

	**Average Discharge**	**Average Sending Duration**
*L1*	0.384966 μAh	24.965 ms
*L2*	0.322929 μAh	22.023 ms
*L3*	0.344028 μAh	22.016 ms

**Table 2.** Energy statistics *L1*–*L3* for measurement 2.

	**Average Discharge**	**Average Sending Duration**
*L1*	0.385638 μAh	24.973 ms
*L2*	0.336713 μAh	22.021 ms
*L3*	0.349736 μAh	22.017 ms

**Table 3.** Energy statistics *L1*–*L3* for measurement 3.

	**Average Discharge**	**Average Sending Duration**
*L1*	0.390695 μAh	24.907 ms
*L2*	0.334943 μAh	22.021 ms
*L3*	0.350460 μAh	22.014 ms

**Table 4.** Data transmission statistics for *L1*.

	**Data Transmitted in Total**	**Packets**
*L1* (run 1)	9.43 MiB	152,119
*L1* (run 2)	9.57 MiB	154,362
*L1* (run 3)	9.52 MiB	153,640
*L1* Min.	9.43 MiB	152,119
*L1* Max.	9.57 MiB	154,362
*L1* Average	9.51 MiB	153,374

**Table 5.** Data transmission statistics for *L2*.

	**Data Transmitted in Total**	**Packets**
*L2* (run 1)	3.82 MiB	154,166
*L2* (run 2)	3.87 MiB	156,048
*L2* (run 3)	3.85 MiB	155,075
*L2* Min.	3.82 MiB	154,166
*L2* Max.	3.87 MiB	156,048
*L2* Average	3.85 MiB	155,096

**Table 6.** Data transmission statistics for *L3*.

	**Data Transmitted in Total**	**Packets**
*L3* (run 1)	3.67 MiB	154,124
*L3* (run 2)	3.71 MiB	155,725
*L3* (run 3)	3.72 MiB	156,217
*L3* Min.	3.67 MiB	154,124
*L3* Max.	3.72 MiB	156,217
*L3* Average	3.70 MiB	155,355
